# Evaluating the Quality of Reporting in Randomized Controlled Trial Abstracts on Diabetic Foot: A Cross-Sectional Study with Interrupted Time-Series Analysis

**DOI:** 10.3390/biomedicines14091943

**Published:** 2026-08-29

**Authors:** Luciana Tonkic, Marin Rudan, Tanja Milicevic Milardovic, Dubravka Vukovic, Ana Sanader Vucemilovic, Doris Rusic, Josipa Bukic

**Affiliations:** 1 Department of Internal Medicine, Division of Endocrinology, Diabetology and Metabolism, University Hospital of Split, 21000 Split, Croatia; tonkicluciana@gmail.com (L.T.); tanja.milicevic2@gmail.com (T.M.M.); 2 Faculty of Dental Medicine and Health, Josip Juraj Strossmayer University of Osijek, 31000 Osijek, Croatia; mrudan@fdmz.hr; 3 Department of Dermatovenerology, University Hospital of Split, 21000 Split, Croatia; ana.sanader@hotmail.com; 4 Department of Pharmacy, University of Split School of Medicine, 21000 Split, Croatia; drusic@mefst.hr (D.R.); jbukic@mefst.hr (J.B.); 5 Department of Laboratory Medicine and Pharmacy, Faculty of Medicine, Josip Juraj Strossmayer University of Osijek, 31000 Osijek, Croatia

**Keywords:** diabetic foot, CONSORT for abstracts, reporting quality, randomized controlled trial, meta-research, interrupted time series

## Abstract

**Background/Objectives**: The aim of this study was to assess the extent to which published abstracts of randomized controlled trials on diabetic foot complied with the Consolidated Standards of Reporting Trials for Abstracts (CONSORT-A) guidelines and to identify factors associated with the quality of reporting. **Methods**: A cross-sectional observational study was designed using the MEDLINE/PubMed database, and the timeframe from 1994 to 2026 was covered by this review. In total, 606 abstracts of randomized controlled trials (RCTs) focusing on diabetic foot were assessed for compliance with the CONSORT-A checklist criteria. **Results**: Ratio of non-pharmacological and pharmacological studies was approximately 1:1. Most of the included abstracts did not report a multicenter design (84.3%) and reported less than one hundred participants included (78.9%). The median total score was six of 17 items (35.29%, IQR 29.41–47.06%). The objective of the study, conclusion and intervention had been reported by over ninety percent of the studies. Randomization method (3.6%), funding (4.8%), recruitment process (6.8%) and numbers of analyzed participants in the study (7.4%) were all reported by less than ten percent of the abstracts. Following CONSORT-A, in 2008, median abstract reporting score improved from 35.29% to 41.18% (*p* < 0.001). **Conclusions**: However, segmented regression showed a secular trend of +0.63 percentage points per year (95% CI 0.14–1.12, *p* = 0.012) with no change in level (*p* = 0.261) or slope (*p* = 0.278) at 2008, indicating that the improvement is not attributable to the guideline itself.

## 1. Introduction

Diabetic foot disease, encompassing diabetic foot ulceration (DFU), peripheral neuropathy, and lower-extremity arterial disease, represents one of the most severe, debilitating, and costly complications of diabetes mellitus [1]. Driven by the continuous global surge in diabetes prevalence, approximately 589 million adults aged 20–79 years are living with diabetes, a figure projected to increase to 853 million by 2050. The incidence of diabetic foot complications has escalated substantially on a global scale [2,3]. Diabetic foot ulcers carry an alarmingly high risk of recurrence, infection, and lower-limb amputation, imposing an immense clinical and financial burden on patients, caregivers, and healthcare delivery systems alike [4,5]. Consequently, there has been a significant expansion in clinical research and randomized controlled trials (RCTs) evaluating novel surgical techniques, pharmacological therapies, bioengineered wound dressings, and advanced offloading devices aimed at accelerating healing and preventing limb loss [6,7].

Randomized controlled trials remain widely regarded as the gold standard for assessing therapeutic efficacy and safety in clinical management. However, the translation of clinical trial evidence into daily clinical practice relies heavily on the transparency, accuracy, and methodological completeness of published reports [8,9]. For the majority of clinicians, the abstract of a journal article serves as the primary source of information accessed during screening of the literature, evidence synthesis, and point-of-care decision-making [10,11]. Incomplete or selective reporting in abstracts can mislead readers regarding a trial’s true validity, overestimate treatment effects, and ultimately jeopardize patient safety [11,12].

To mitigate these reporting deficiencies, the Consolidated Standards of Reporting Trials for Abstracts (CONSORT-A) guidelines were introduced in 2008 as an official extension of the main CONSORT statement [13,14]. The CONSORT-A framework consists of a standardized 17-item checklist designed to ensure transparent and structured reporting of essential methodological and clinical details within wordcount constraints [13,14].

These 17 specific items span all structural sections of an abstract: the title requiring identification of the trial design (Item 1), contact details for the corresponding authors (Item 2), explicit descriptions of the trial design (Item 3), participant eligibility criteria and settings (Item 4), interventions (Item 5), objectives or hypotheses (Item 6), primary outcomes (Item 7), sequence generation (Item 8), allocation concealment (Item 9), blinding procedures (Item 10), the results section detailing numbers randomized (Item 11), recruitment status (Item 12), numbers analyzed (Item 13), primary outcome effect sizes with precision measures (Item 14), important harms (Item 15), and finally, a balanced conclusion (Item 16) alongside trial registration and funding disclosures (Item 17) [13,14].

Adherence to CONSORT-A guidelines is particularly crucial in complex fields such as diabetic foot care, where high patient heterogeneity, variable wound classification systems, diverse outcome measures, and elevated risk of bias necessitate uncompromising reporting clarity [6,7,15]. Despite the widespread endorsement of CONSORT guidelines by major biomedical journals, empirical evaluations across various medical specialties consistently indicate that the reporting quality in trial abstracts remains suboptimal [12,16].

The aim of this study was to assess the extent to which published abstracts of randomized controlled trials on diabetic foot complied with CONSORT-A guidelines and to identify factors associated with reporting quality over time. We hypothesized that the overall reporting quality of diabetic foot RCT abstracts remains suboptimal. Furthermore, we postulated that higher CONSORT-A compliance scores are positively associated with a more recent publication year, a higher journal impact factor, and explicit editorial endorsement of CONSORT guidelines.

## 2. Materials and Methods

### 2.1. Study Design and Search Strategy

To evaluate the reporting quality of randomized controlled trials (RCTs) focused on diabetic foot, a cross-sectional observational study was designed. The MEDLINE/PubMed database was chosen as the primary source for data retrieval. Only trials structured with a distinct control group were included in the analysis. Within these included studies, the main intervention was compared against a placebo, an active treatment, or no treatment at all. The study is reported in accordance with the STROBE statement, adapted as appropriate for methodological research.

During the screening phase, strict exclusion criteria were applied. Observational studies, along with any other non-RCT designs, were entirely omitted. Furthermore, papers were excluded if an abstract was not provided, if the research was not conducted on humans, or if diabetic foot was not the main clinical focus.

The timeframe from 1994 to 2026 was covered by this review. This specific period was chosen so that abstract quality could be compared before and after the 2008 publication of the CONSORT guidelines. The starting year reflects the introduction of the MeSH term “Diabetic Foot” (D017719) in 1994; before that date, relevant trials were indexed under Diabetes Mellitus, Foot Diseases or Foot Ulcer, so a MeSH-based search cannot reliably retrieve earlier records.

To retrieve the relevant literature, the following exact syntax was entered into MEDLINE/PubMed: (“diabetic foot”[MeSH Terms] AND (randomized controlled trial[Filter])). A complete inventory of the extracted abstracts, together with the item-level scoring manual, can be provided to interested parties upon reasonable request.

The initial search retrieved a total of 746 records. During title and abstract screening, 140 records were excluded: 98 did not employ a randomized controlled design, 22 did not address diabetic foot as the primary topic, and 20 had no abstract available. The remaining 606 abstracts of randomized controlled trials on diabetic foot constituted the final sample that was assessed for adherence to the CONSORT-A checklist.

### 2.2. Data Extraction and Scoring

The extracted abstracts were independently evaluated by two reviewers. This assessment was strictly guided by the established CONSORT framework. Each of the 17 CONSORT-A items was scored dichotomously (1 = item adequately reported, 0 = item not reported or reported incompletely); partial credit was not awarded. A written scoring manual specifying the operational rule applied to each item was used throughout and is provided as Supplementary Materials. The screening team was composed of an endocrinology specialist with clinical trial experience (L.T.) and a research methodologist whose previous work focused heavily on CONSORT compliance (D.R.). When discrepancies in scoring were encountered, they were resolved through structured discussion. These arbitrations were overseen by a third, senior investigator who is highly experienced in both RCT execution and reporting standards (J.B.). The dataset analyzed here is the post-consensus dataset; inter-rater agreement was calculated on the two independent pre-consensus datasets.

All items and all study characteristics were coded exclusively from the abstract text as displayed in MEDLINE/PubMed; full texts were not consulted. Consequently, a value of 0 indicates that a characteristic was not stated in the abstract rather than that it was absent from the trial, and the categories in Tables 2, 5 and 6 are labeled accordingly. Journal impact factor and journal quartile were assigned from a single, most recent Journal Citation Reports release, rather than from the release corresponding to each year of publication, because quartile rankings are not available for the earlier part of the study period; these two variables are therefore correlated with publication year, which is addressed by including publication year in the multivariate model.

Interventions were classified into ten mutually exclusive categories. The taxonomy was developed inductively: the free-text intervention description of every included abstract was reviewed and grouped into provisional categories, which were then consolidated into the final ten-category scheme by consensus between the assessors. Each abstract was assigned to a single category.

### 2.3. Statistical Analysis

Basic descriptive statistics were used to present the findings. Overall numbers and proportions were calculated for categorical data. For continuous variables, medians with their interquartile ranges (IQRs) were reported, alongside means paired with either the standard deviation (SD) or a 95% confidence interval (CI). To determine the level of consistency between the independent reviewers, the Cohen κ coefficient was calculated. Interobserver agreement was considered acceptable when κ was higher than 0.6. Agreement was additionally interpreted according to the categories of Landis and Koch. Because a preliminary Shapiro–Wilk test indicated that the data were not normally distributed, the non-parametric Mann–Whitney U test was applied. This test was used to contrast quality scores between the pre-2008 and post-2008 periods. To identify specific factors that impacted overall reporting quality, a univariate regression analysis was initially performed. All study characteristics were subsequently entered into a single prespecified multivariate regression model, rather than only those significant in the univariate step, in order to avoid bias in the coefficients and confidence intervals; publication year was included as a covariate in order to distinguish the contribution of individual characteristics from the underlying temporal trend. Regression estimates are expressed in percentage points of the maximum attainable score. Model assumptions were checked on the residuals of the full model: the Shapiro–Wilk test was not significant (*p* = 0.267), whereas the Breusch–Pagan test indicated heteroscedasticity (*p* = 0.002), and heteroscedasticity-consistent (HC3) standard errors were therefore used. Multicollinearity was assessed by variance inflation factors. Because a comparison of two periods cannot distinguish a guideline effect from an underlying trend, a segmented (interrupted time-series) regression was additionally fitted, modeling the pre-2008 slope, the change in level at 2008 and the change in slope thereafter; abstracts published during 2008 were assigned to the post-guideline period. Reporting of each individual item was compared between the two periods by the χ^2^ test, with the Benjamini–Hochberg procedure applied across the 17 items. Journal quartile could not be assigned for two journals, so analyses involving quartile are based on 604 abstracts. All statistical procedures were carried out using IBM SPSS Statistics for Windows, Version 25.0 (IBM Corp., Armonk, NY, USA). Regression models, robust standard errors, segmented regression and the multiplicity adjustment were computed and independently verified in Python 3 (Python Software Foundation, Beaverton, OR, USA, https://www.python.org, specifically utilizing the statsmodels module, Version 0.14). The threshold for statistical significance was defined as *p* < 0.05.

## 3. Results

Agreement between the two reviewers was quantified using Cohen’s kappa statistic applied to each CONSORT-A item individually. Every calculated value met or exceeded the accepted minimum of 0.60, supporting the reliability of the scoring process, as shown in Table 1. Interpreted according to Landis and Koch, agreement was almost perfect for five items, substantial for ten and moderate for two (participants, κ 0.608; numbers randomized, κ 0.602); four values lie close to the 0.60 threshold, and the corresponding items should be interpreted with caution.

The characteristics of included RCT abstracts are demonstrated in Table 2. The ratio of non-pharmacological and pharmacological studies was approximately 1:1 (325, 53.6% and 281, 46.4%, non-pharmacological studies and pharmacological studies). Most of the included abstracts did not report a multicenter design (511/606, 84.3%), and there was a considerably higher proportion of studies with less than one hundred participants included (478/606, 78.9%) compared to those which had one hundred or more participants (128/606, 21.1%). More than two fifths of reported studies were published in journals from the first quartile (246/606, 40.6%). The median journal impact factor was 2.60 (1.50–3.70 IQR), and the included studies had 6.63 authors on average.

As per Table 3, the most frequently reported items in the abstracts included were the objective of the study (96.7%) and the conclusion of the study (96.7%), followed by intervention of the study (93.1%). Those were the only three items which had been reported by over ninety percent of the studies. The outcome of the study was also correctly reported in the methods section of the abstract by most of the abstracts (72.6%).

Description of the way randomization was conducted was the worst reported item from the checklist, with only twenty-two studies reporting it correctly (3.6%). Funding (4.8%), recruitment process (6.8%) and numbers of analyzed participants in the study (7.4%) were all reported by less than ten percent of the abstracts.

As seen in Table 4, the median total score was six (IQR 5–8) of a maximum of 17 points, or 35.29% (IQR 29.41–47.06%). The highest score was 14 out of 17 (82.35%), while the lowest was one out of 17 (5.88%).

The highest mean score was described in studies which reported industry funding (54.25%, CI 46.70–61.80). Studies that were published in journals belonging to the fourth quartile had the lowest mean score of all analyzed groups (30.68%, CI 28.22–33.14), as seen in Table 5, which also reports group sizes and *p*-values for each comparison.

Separate univariate regressions for each characteristic against the overall reporting score were conducted. All characteristics were significantly associated with the total score (*p* < 0.01) except the type of intervention (*p* = 0.215) and whether the journal belonged to the second quartile (*p* = 0.594). Cut-off points for the number of authors and journal impact factor were set on median values of those variables. All study characteristics, and not only those significant in the univariate step, were subsequently carried as predictors in the multivariate regression.

As seen in Table 6. Multivariate modeling identified seven independent predictors: structured abstract, industry funding, hospital setting, multicenter study, study with one hundred or more participants, journal impact factor exceeding 2.60 and year of publication (adjusted R^2^ = 0.321, explaining 32.1% of score variance, *n* = 604). Journal belonging to third and fourth quartile was associated with lower scores. Significance of results and authorship exceeding six contributors were significant in univariate analysis but no longer after adjustment for year of publication, indicating that these associations were confounded by time. Variance inflation factors were below two for all predictors, including impact factor and journal quartile.

As Table 7 demonstrates, among types of interventions, advanced physical and energy-based therapies were reported the most among included studies (127, 21.0%), followed by wound dressings and topical agents (109, 18.0%). The least number of studies were reported for surgical groups and procedural interventions (12, 2.0%) and for temperature and pressure monitoring (8, 1.3%).

**Figure 1:**
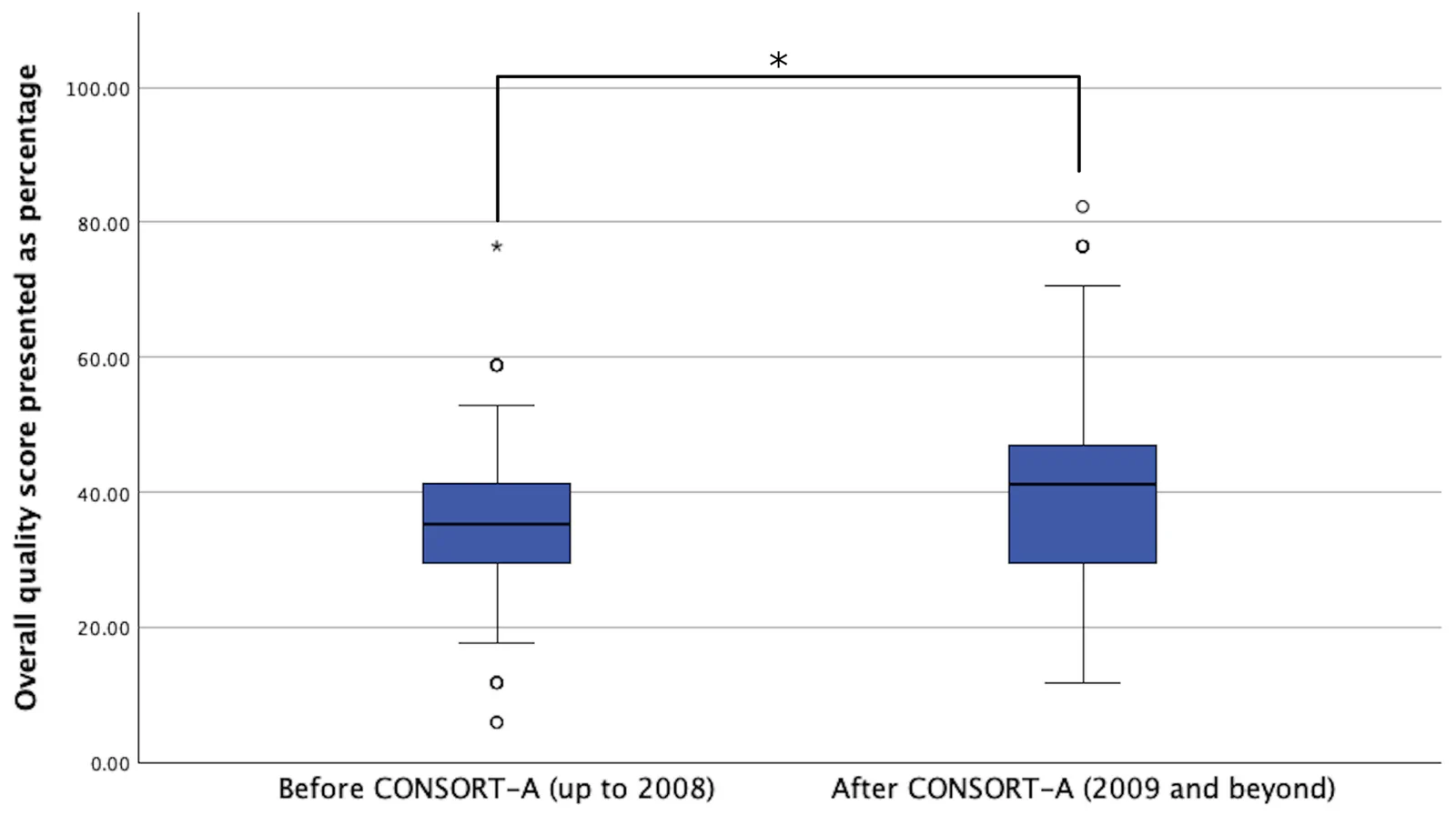
Overall CONSORT-A reporting quality score of diabetic foot RCT abstracts before and after publication of CONSORT-A Guidelines (2008) [13], * *p* < 0.001.

With 2008 marking the introduction of CONSORT-A, the dataset was split accordingly and reporting scores compared across the two eras. Pre-guideline abstracts (*n* = 115) had a median score of 35.29% (IQR 29.41–41.18%), whereas post-2008 publications (*n* = 491) scored notably higher at 41.18% (IQR 29.41–47.06%). The improvement, though modest in absolute terms, was statistically significant (*p* < 0.001), as shown in Figure 1.

This dichotomous comparison does not, however, account for the underlying temporal trend in reporting quality. In segmented (interrupted time-series) regression, the pre-2008 slope was positive and significant (+0.63 percentage points per year, 95% CI 0.14–1.12, *p* = 0.012), whereas neither the change in level at 2008 (−2.54 percentage points, 95% CI −6.97 to 1.89, *p* = 0.261) nor the change in slope thereafter (−0.29, 95% CI −0.82 to 0.24, *p* = 0.278) reached statistical significance. Consistent with this, the Mann–Whitney comparison remained significant when the sample was divided at a series of arbitrary alternative years (2003, 2005, 2010, 2013, 2015 and 2018; all *p* < 0.001). Taken together, these analyses indicate a gradual secular improvement in reporting quality rather than a discrete change coinciding with the publication of CONSORT-A.

At the level of individual checklist items (Table 8), reporting improved significantly after 2008 for four of the seventeen items: title (34.8% to 60.7%), trial registration (3.5% to 23.4%), interventions (87.0% to 94.5%) and outcome reported in the Methods section of the abstract (61.7% to 75.2%). Reporting of funding rose from 0% to 5.9% but did not reach significance after adjustment for multiple comparisons (adjusted *p* = 0.052). The items most closely related to risk of bias did not change: method of randomization (3.5% to 3.7%), blinding (20.0% to 23.0%) and numbers analyzed (7.0% to 7.5%).

Finally, the internal consistency between the claims made in the abstracts and the data supporting them was examined. A benefit of the intervention was claimed in 353 abstracts (58.3%), whereas the primary outcome was reported together with an effect estimate and a measure of precision in only 90 abstracts (14.9%). Accordingly, 293 abstracts (48.3%) asserted a treatment benefit that a reader could not verify from the quantitative data presented in the abstract. Because our coding recorded the claim made in the abstract rather than a verified statistical result, we could not distinguish genuinely significant findings from selectively framed ones; a formal assessment of spin would require evaluation against the full texts.

In the boxplot of the overall CONSORT-A reporting quality score (expressed as percentage of the maximum of 17 items) for abstracts published before 2008 (*n* = 115) and in or after 2008 (*n* = 491), the box denotes the interquartile range, the horizontal line denotes the median, the whiskers denote values within 1.5 × IQR of the quartiles, and individual points denote values beyond that range. * *p* < 0.001, Mann–Whitney U test.

## 4. Discussion

This study analyzed 606 abstracts of randomized controlled trials (RCTs) focusing on diabetic foot, with the aim of assessing their compliance with the CONSORT-A checklist criteria.

Since 1987, the ad hoc Working Group for the Critical Appraisal of Medical Literature has recommended the use of informative abstracts [10].

The 2008 CONSORT for Abstracts framework aimed to ensure that readers receive a transparent, structured, and methodologically sound summary of an RCT, enabling them to evaluate the trial’s validity even when full text access is limited [11,13]. Recently, the CONSORT Group published the updated CONSORT 2025 Statement, which restructured the primary checklist into 30 items, introduced seven new items, and established a dedicated section on Open Science to enhance transparency and reproducibility. Notably, while this comprehensive update addresses main trial reports, the CONSORT Group recommends the continued use of the existing 2008 extension for abstracts until an aligned update is formally released [9].

Consequently, our choice of the CONSORT 2008 for Abstracts tool remains fully appropriate and aligned with current methodological recommendations.

The analyses revealed that the proportion of pharmacological and non-pharmacological studies investigating diabetic foot disease was equal. Most abstracts did not report a hospital setting or a multicenter design and involved less than 100 participants. It is noteworthy that the majority of articles reported statistically significant results and presented a clearly stated study conclusion. However, given that funding information was disclosed in less than 10% of the studies, these findings warrant careful consideration. Funding is a factor that can substantially influence clinical judgment and the likelihood of positive outcomes in randomized controlled trials despite the expectation that such studies should remain objective and unbiased [17,18,19,20]. Alongside funding, participant flow and sample size were reported with similarly low frequencies in our study. Nevertheless, randomization emerged as the most poorly reported item, being mentioned in only 3.6% of the abstracts.

Unfortunately, these findings are not surprising. The findings of Ghimire et al. highlight persistent deficiencies in adherence to the CONSORT for Abstracts guidelines, particularly in the reporting of key methodological aspects, underscoring the need for improved reporting standards in RCT abstracts [12].

Our study, however, stands out positively in terms of the proportion of non-pharmacological interventions, which were represented in an equal ratio to pharmacological interventions (1:1). In contrast, Germini et al., in studies conducted within the field of emergency medicine, demonstrated that pharmacological interventions significantly outnumbered non-pharmacological interventions in other research areas [21].

Following the introduction of CONSORT-A in 2008, median abstract reporting scores significantly improved from 35.29% to 41.18% (*p* < 0.001). However, segmented regression showed a significant underlying trend in reporting quality but no significant change in level or slope at 2008, indicating that this improvement reflects a gradual trend rather than an effect of the guideline itself. Efforts to improve the quality of RCT abstracts are not new. The implementation of the CONSORT-A checklist by journal editors has become increasingly common. Hopewell et al. specifically evaluated the impact of endorsing CONSORT-A guidelines and reported the expected finding that their adoption leads to improved reporting quality in abstracts of RCTs [14]. 

Industry funding was the strongest single predictor of reporting quality in our study (+9.65 percentage points after adjustment). As all variables were coded from the abstract, this finding should be understood as a characteristic of reporting rather than of funding itself. Abstracts which declare the source of funding also tend to report other checklist items more completely, which could be explained by the involvement of professional medical writers and by regulatory reporting requirements in industry-sponsored trials. A similar explanation applies to hospital setting (+5.57 percentage points), which probably marks abstracts that describe participants and setting in more detail. Structured abstracts scored 4.81 percentage points higher, which supports the view that journal formatting requirements are among the more effective tools available to editors.

Two previous studies used the same methodology as our study. Vrebalov Cindro et al. assessed abstracts of randomized controlled trials on Helicobacter pylori infection [20], while Vucinovic et al. assessed glaucoma trial abstracts [20]. Our results are in line with both, as overall adherence was low and structured abstract format was associated with better reporting. Our study adds to this work by separating the effect of the guideline from the underlying temporal trend, which was not done in these studies and which suggests that improvements reported there could also be partly explained by a general trend over time.

Indeed, many high-impact journals have endorsed the CONSORT Statement and the CONSORT-A checklist as essential tools for improving the transparency and completeness of trial reporting.

As previously discussed, contemporary research findings play a crucial role in guiding clinical practice and informing the design of future studies. Therefore, accurate and comprehensive reporting is of paramount importance. Our findings confirm those of previous investigations, demonstrating that the overall quality of reporting in RCT abstracts remains inadequate. Greater adherence to CONSORT-A guidelines, encouraged through stronger endorsement and enforcement by scientific journals, may help improve reporting standards in the future [7,16,20].

### Limitations

First, the MeSH term “Diabetic Foot” was introduced only in 1994, so trials published before that year could not be retrieved; the pre-2008 group is therefore restricted to 1994–2007 and comprises 115 abstracts. Second, by evaluating abstracts alone, we were unable to determine whether the corresponding full-text studies adequately fulfilled all methodological and reporting requirements. Some studies may have met the necessary standards but failed to report them appropriately within the abstract due to poor structure or non-compliance with CONSORT-A recommendations. Third, all study characteristics were coded from the abstract, so categories such as “no industry funding declared” or “multicenter design not stated” reflect the absence of a statement rather than the absence of the feature itself. Fourth, journal impact factor and quartile were assigned from a single Journal Citation Reports release and are therefore correlated with publication year. Finally, an important limitation is that our sample consisted exclusively of RCTs indexed in PubMed. Therefore, relevant studies published in other databases or within the gray literature may not have been captured by our analysis.

## 5. Conclusions

Our study aims to contribute to the growing body of publications highlighting the importance of high-quality reporting in clinical research. The median abstract reported only six of 17 CONSORT-A items (35.29%), and reporting quality improved gradually over the study period rather than as a consequence of the guideline itself. Greater adherence to CONSORT-A recommendations and their inclusion in author guidelines across scientific journals could improve reporting quality, facilitate the efficient dissemination and interpretation of research findings, and ultimately support better clinical decision-making and patient care. We believe that abstracts should provide a clear, accurate, and comprehensive summary of available evidence, enabling clinicians and researchers to effectively apply existing knowledge while fostering the development of future research.

## Figures and Tables

**Table 1:** Inter-rater reliability of CONSORT-A checklist scoring in diabetic foot RCT abstracts.

Segment	Kappa	Strength of Agreement (Landis & Koch)
Title	0.919	Almost perfect
Authors	0.969	Almost perfect
Trial design	0.734	Substantial
Methods		
Participants	0.608	Moderate
Intervention	0.848	Almost perfect
Objective	0.704	Substantial
Outcome	0.629	Substantial
Randomization	0.704	Substantial
Blinding	0.617	Substantial
Results		
Numbers randomized	0.602	Moderate
Recruitment	0.631	Substantial
Numbers analyzed	0.620	Substantial
Outcome	0.660	Substantial
Harms	0.911	Almost perfect
Conclusions	0.681	Substantial
Trial registration	0.877	Almost perfect
Funding	0.655	Substantial

**Table 2:** Characteristics of the reviewed diabetic foot RCT abstracts, as reported in the abstract text.

Characteristics	*n*	%
Type of intervention		
Non-pharmacological	325	53.6
Pharmacological	281	46.4
Multicenter design		
Multicenter design not stated	511	84.3
Multicenter design stated	95	15.7
Number of participants		
<100	478	78.9
≥100	128	21.1
Claimed benefit		
Benefit not claimed	253	41.7
Benefit claimed	353	58.3
Setting		
Hospital setting not stated	544	89.8
Hospital setting stated	62	10.2
Abstract structure		
Unstructured abstract	209	34.5
Structured abstract	397	65.5
Declared funding source		
No industry funding declared	588	97.0
Industry funding declared	18	3.0
Journal quartile		
1st	246	40.6
2nd	152	25.1
3rd	141	23.3
4th	65	10.7
Not assigned	2	0.3
	Mean (SD)	Median (IQR)
Number of authors	6.63 (3.57)	6.00 (4.00–8.00)
Impact factor	4.49 (7.81)	2.60 (1.50–3.70)

**Table 3:** Individual CONSORT-A item reporting rates in diabetic foot RCT abstracts.

Segment	*n*	%
Title	338	55.8
Authors	238	39.3
Trial design	200	33.0
Methods		
Participants	44	7.3
Intervention	564	93.1
Objective	586	96.7
Outcome	440	72.6
Randomization	22	3.6
Blinding	136	22.4
Results		
Numbers randomized	353	58.3
Recruitment	41	6.8
Numbers analyzed	45	7.4
Outcome	90	14.9
Harms	111	18.3
Conclusions	586	96.7
Trial registration	119	19.6
Funding	29	4.8

**Table 4:** Summary of overall reporting quality scores in diabetic foot RCT abstracts.

	Score	Score (%)
Mean	6.50	38.26
SD	2.04	12.01
95% CI	6.34–6.67	37.31–39.22
Median	6	35.29
IQR	5–8	29.41–47.06

**Table 5:** Mean CONSORT-A adherence score by study characteristic, with group sizes and *p*-values (Mann–Whitney U test; Kruskal–Wallis test for journal quartile).

Characteristics	*n*	Mean Score (%)	95% CI	*p*
Type of intervention				
Non-pharmacological	325	37.70	36.40–39.01	0.127
Pharmacological	281	38.92	37.51–40.32	
Multicenter design				
Not stated	511	36.72	35.75–37.70	<0.001
Stated	95	46.56	44.01–49.12	
Number of participants				
<100	478	36.38	35.43–37.33	<0.001
≥100	128	45.31	42.84–47.78	
Claimed benefit				
Not claimed	253	36.34	34.72–37.96	<0.001
Claimed	353	39.64	38.50–40.79	
Hospital setting				
Not stated	544	37.22	36.27–38.17	<0.001
Stated	62	47.44	43.88–50.99	
Abstract structure				
Unstructured abstract	209	33.86	32.35–35.37	<0.001
Structured abstract	397	40.58	39.42–41.75	
Declared funding source				
No industry funding declared	588	37.78	36.84–38.71	<0.001
Industry funding declared	18	54.25	46.70–61.80	
Journal quartile				
1st	246	40.99	39.43–42.54	<0.001
2nd	152	40.33	38.47–42.18	
3rd	141	34.79	33.13–36.46	
4th	65	30.68	28.22–33.14	
Number of authors				
≤6	357	36.40	35.26–37.53	<0.001
>6	249	40.94	39.33–42.55	
Impact factor				
≤2.60	309	35.16	34.00–36.33	<0.001
>2.60	295	41.52	40.07–42.97	

**Table 6:** Predictors of the overall CONSORT-A adherence score: univariate and pre-specified multivariable linear regression.

Characteristics	Univariate Analysis, Estimate (95% CI)	Multivariable Analysis, Estimate (95% CI)
Type of intervention		
Non-pharmacological	Ref.	Ref.
Pharmacological	1.21 (−0.71–3.13)	1.03 (−0.59–2.65)
Multicenter design		
Not stated	Ref.	Ref.
Stated	9.84 (7.09–12.59) ***	4.19 (1.37–7.02) **
Number of participants		
<100	Ref.	Ref.
≥100	8.94 (6.28–11.59) ***	3.84 (1.34–6.34) **
Claimed benefit		
Not claimed	Ref.	Ref.
Claimed	3.30 (1.32–5.29) **	1.29 (−0.44–3.02)
Hospital setting		
Not stated	Ref.	Ref.
Stated	10.22 (6.51–13.93) ***	5.57 (2.55–8.59) ***
Abstract structure		
Unstructured abstract	Ref.	Ref.
Structured abstract	6.72 (4.81–8.64) ***	4.81 (3.05–6.57) ***
Declared funding source		
No industry funding declared	Ref.	Ref.
Industry funding declared	16.47 (8.65–24.30) ***	9.65 (3.38–15.92) **
Journal quartile		
1st	Ref.	Ref.
2nd	−0.66 (−3.09–1.77)	0.35 (−1.89–2.59)
3rd	−6.19 (−8.48–−3.91) ***	−2.76 (−5.39–−0.13) *
4th	−10.31 (−13.23–−7.38) ***	−5.81 (−8.93–−2.69) ***
Number of authors		
≤6	Ref.	Ref.
>6	4.54 (2.57–6.51) ***	1.48 (−0.26–3.21)
Impact factor		
≤2.60	Ref.	Ref.
>2.60	6.36 (4.49–8.22) ***	2.85 (0.66–5.04) *
Year of publication (per year)	0.32 (0.21–0.44) ***	0.26 (0.15–0.36) ***

Estimates are expressed in percentage points of the maximum attainable CONSORT-A score (17 items) and were obtained by linear regression with heteroscedasticity-consistent (HC3) standard errors. The multivariable model is a single pre-specified model containing all listed characteristics (*n* = 604; adjusted R^2^ = 0.321). * *p* < 0.05, ** *p* < 0.01, *** *p* < 0.001.

**Table 7:** Intervention types investigated in included diabetic foot RCT abstracts.

Intervention Type	*n*	%
Advanced physical and energy-based therapies	127	21.0
Wound dressings and topical agents	109	18.0
Biological and regenerative therapies	92	15.2
Systemic pharmacological therapies	92	15.2
Education, self-care and behavioral interventions	62	10.2
Offloading and orthotic devices	47	7.8
Topical pharmacological therapy	36	5.9
Exercise and physical rehabilitation	21	3.5
Surgical and procedural interventions	12	2.0
Temperature and pressure monitoring	8	1.3

**Table 8:** Reporting of individual CONSORT-A items before and after publication of CONSORT-A (2008) [13].

CONSORT-A Item	Before 2008 (*n* = 115), %	2008 Onwards (*n* = 491), %	*p*	*p* (Benjamini–Hochberg)
Title	34.8	60.7	<0.001	<0.001
Authors	44.3	38.1	0.258	0.398
Trial design	38.3	31.8	0.222	0.377
Participants	2.6	8.4	0.053	0.150
Interventions	87.0	94.5	0.008	0.033
Objective	95.7	97.0	0.683	0.829
Outcome (methods)	61.7	75.2	0.005	0.030
Randomization	3.5	3.7	1.000	1.000
Blinding	20.0	23.0	0.567	0.741
Numbers randomized	51.3	59.9	0.116	0.281
Recruitment	3.5	7.5	0.176	0.333
Numbers analyzed	7.0	7.5	0.988	1.000
Outcome (results)	13.9	15.1	0.866	0.982
Harms	23.5	17.1	0.145	0.309
Conclusions	94.8	97.2	0.323	0.457
Trial registration	3.5	23.4	<0.001	<0.001
Funding	0.0	5.9	0.015	0.052

## Data Availability

The full extracted dataset and the item-level scoring manual are available upon request to the corresponding author.

## Supplementary Materials

The following supporting information can be downloaded at: https://www.mdpi.com/article/10.3390/biomedicines14091943/s1, File S1: Scoring Manual and STROBE Checklist. References [11,13,22,23] are cited in the supplementary materials.

## References

[1] Armstrong D.G., Boulton A.J.M., Bus S.A. (2017). Diabetic Foot Ulcers and Their Recurrence. N. Engl. J. Med..

[2] Genitsaridi I., Salpea P., Salim A., Sajjadi S.F., Tomic D., James S., Thirunavukkarasu S., Issaka A., Chen L., Basit A. (2025). 11th edition of the IDF Diabetes Atlas: Global, regional, and national diabetes prevalence estimates for 2024 and projections for 2050. Lancet Diabetes Endocrinol..

[3] Zhang P., Lu J., Jing Y., Tang S., Zhu D., Bi Y. (2017). Global epidemiology of diabetic foot ulceration: A systematic review and meta-analysis. Ann. Med..

[4] Lazzarini P.A., Pacella R.E., Armstrong D.G., van Netten J.J. (2018). Diabetes-related lower-extremity complications are a leading cause of the global burden of disability. Diabet. Med..

[5] McDermott K., Fang M., Boulton A.J.M., Selvin E., Hicks C.W. (2023). Etiology, Epidemiology, and Disparities in the Burden of Diabetic Foot Ulcers. Diabetes Care.

[6] Schaper N.C., van Netten J.J., Apelqvist J., Bus S.A., Hinchliffe R.J., Lipsky B.A., IWGDF Editorial Board (2020). Practical Guidelines on the prevention and management of diabetic foot disease (IWGDF 2019 update). Diabetes Metab. Res. Rev..

[7] Jeffcoate W.J., Bus S.A., Game F.L., Hinchliffe R.J., Price P.E., Schaper N.C. (2016). Reporting standards of studies and papers on the prevention and management of foot ulcers in diabetes: Required details and markers of good quality. Lancet Diabetes Endocrinol..

[8] Moher D., Hopewell S., Schulz K.F., Montori V., Gøtzsche P.C., Devereaux P.J., Elbourne D., Egger M., Altman D.G. (2010). CONSORT 2010 explanation and elaboration: Updated guidelines for reporting parallel group randomised trials. BMJ.

[9] Hopewell S., Chan A.W., Collins G.S., Hróbjartsson A., Moher D., Schulz K.F., Tunn R., Aggarwal R., Berkwits M., Berlin J.A. (2025). CONSORT 2025 statement: Updated guideline for reporting randomised trials. Lancet.

[10] (1987). Ad Hoc Working Group for Critical Appraisal of the Medical Literature. A proposal for more informative abstracts of clinical articles. Ann. Intern. Med..

[11] Hopewell S., Clarke M., Moher D., Wager E., Middleton P., Altman D.G., Schulz K.F., CONSORT Group (2008). CONSORT for reporting randomised trials in journal and conference abstracts. Lancet.

[12] Catalão M.J., Figueiredo J., Henriques M.X., Gomes J.P., Filipe S.R. (2014). Optimization of Fluorescent Tools for Cell Biology Studies in Gram-Positive Bacteria. PLoS ONE.

[13] Hopewell S., Clarke M., Moher D., Wager E., Middleton P., Altman D.G., Schulz K.F., CONSORT Group (2008). CONSORT for reporting randomized controlled trials in journal and conference abstracts: Explanation and elaboration. PLoS Med..

[14] Hopewell S., Ravaud P., Baron G., Boutron I. (2012). Effect of editors’ implementation of CONSORT guidelines on the reporting of abstracts in high impact medical journals: Interrupted time series analysis. BMJ.

[15] Game F.L., Attinger C.E., Hartemann A., Hinchliffe R.J., Londahl M., Price P.E., Jeffcoate W.J. (2016). IWGDF guidance on use of interventions to enhance the healing of chronic ulcers of the foot in diabetes. Diabetes Metab. Res. Rev..

[16] Turner L., Shamseer L., Altman D.G., Weeks L., Peters J., Kober T., Dias S., Schulz K.F., Plint A.C., Moher D. (2012). Consolidated standards of reporting trials (CONSORT) and the completeness of reporting of randomised controlled trials (RCTs) published in medical journals. Cochrane Database Syst. Rev..

[17] Als-Nielsen B., Chen W., Gluud C., Kjaergard L.L. (2003). Association of funding and conclusions in randomized drug trials: A reflection of treatment effect or study design?. JAMA.

[18] Lundh A., Lexchin J., Mintzes B., Schroll J.B., Bero L. (2017). Industry sponsorship and research outcome. Cochrane Database Syst. Rev..

[19] Stpiczyńska M., Kamińska M., Davies K.L. (2021). Nectar secretion in a dry habitat: Structure of the nectary in two endangered Mexican species of *Barkeria* (Orchidaceae). PeerJ.

[20] Esmael A., Mihret A., Abebe T., Mussa D., Neway S., Ernst J., Rengarajan J., Wassie L., Howe R. (2022). Persistent expression of activation markers on Mycobacterium tuberculosis-specific CD4 T cells in smear negative TB patients. PLoS ONE.

[21] Germini F., Marcucci M., Fedele M., Galli M.G., Heath T., Mbuagbaw L., Salvatori V., Veronese G., Worster A., Thabane L. (2020). Quality of reporting in abstracts of RCTs published in emergency medicine journals: A systematic survey of the literature suggests we can do better. Emerg. Med. J..

[22] Landis J.R., Koch G.G. (1977). The measurement of observer agreement for categorical data. Biometrics.

[23] von Elm E., Altman D.G., Egger M., Pocock S.J., Gøtzsche P.C., Vandenbroucke J.P., STROBE Initiative (2007). The Strengthening the Reporting of Observational Studies in Epidemiology (STROBE) statement: Guidelines for reporting observational studies. Lancet.

