# Supplementary material for: Evaluating the Quality of Reporting in Randomized Controlled Trial Abstracts on Diabetic Foot: A Cross-Sectional Study with Interrupted Time-Series Analysis
Source: Biomedicines. 2026 Aug 29;14(9):1943. doi: 10.3390/biomedicines14091943 (PMC13604712; doi:10.3390/biomedicines14091943)
Supplement: Supplementary file 1 [file biomedicines-14-01943-s001.zip › biomedicines-4500826-supplementary.pdf]

Supplementary Material S1

CONSORT-A scoring manual

Evaluating the Quality of Reporting in Randomized Controlled Trial Abstracts on Diabetic Foot: A Cross-Sectional Analysis

1. Purpose and scope

This manual specifies the operational rules that were applied when scoring randomized controlled trial (RCT) abstracts on diabetic foot against the CONSORT extension for abstracts (CONSORT-A; Hopewell et al., 2008). It was used by both assessors throughout data extraction and is reproduced here so that the scoring procedure can be replicated. The manual covers the 17 CONSORT-A checklist items and the additional study characteristics that were recorded for each abstract.

2. General scoring rules

Rule	Specification
Unit of assessment	The abstract only. Each item was judged solely on the abstract text as displayed in MEDLINE/PubMed. Full texts, figures, tables and supplementary files were not consulted at any stage.
Scale	Each of the 17 items was scored dichotomously: 1 = the item was adequately reported; 0 = the item was not reported, or was reported only partially or ambiguously. Partial credit was not awarded.
Total score	The sum of the 17 item scores, ranging from 0 to 17. For analysis the total was also expressed as a percentage of the maximum attainable score (score / 17 × 100).
Absence of a statement	Where a characteristic was not mentioned in the abstract, it was coded 0. A score of 0 therefore indicates that the information was not reported, not that the corresponding feature was absent from the trial. This applies both to the checklist items and to the study characteristics in Section 4.
Ambiguity	Where the wording of an abstract left genuine doubt as to whether an item had been reported, the item was scored 0 and the record was flagged for discussion between the assessors.
Language	Abstracts in any language were eligible provided an English abstract was available in MEDLINE/PubMed; scoring was performed on the English abstract.

3. CONSORT-A checklist items

The third column reproduces the item as worded in the CONSORT extension for abstracts; the fourth gives the operational rule that was applied in this study. A score of 1 was awarded only when the stated condition was fully met. Where our rule was more permissive or more demanding than the checklist wording, this is stated in Section 3.1.

No.	Item	CONSORT-A item description	Operational rule applied for a score of 1
1	Title	Identification of the study as randomized.	The title identifies the study as randomized (for example, “randomised”, “randomized”, “randomised controlled trial”).
2	Authors	Contact details for the corresponding author.	A contact e-mail address for at least one author is available in the MEDLINE/PubMed record (visible after expanding the author affiliation details). See note (a) below.
3	Trial design	Description of the trial design.	The abstract states the trial design using at least one of the following descriptors: parallel group, cluster randomized, crossover, factorial, superiority, equivalence or non-inferiority.
4	Participants	Eligibility criteria for participants and the settings where the data were collected.	Both elements are stated: the eligibility criteria for participants and the specific setting in which the data were collected (for example, a named hospital or clinic). A reference to a country, or to “a hospital” without further specification, was not sufficient.
5	Interventions	Interventions intended for each group.	The interventions intended for both the experimental group (for example, the drug or procedure under study) and the control group (for example, placebo, active comparator or no treatment) are described.
6	Objective	Specific objective or hypothesis.	The objective or specific hypothesis of the trial is clearly stated.

7	Outcome	Clearly defined primary outcome for this report.	The primary outcome of the trial is stated in the Methods part of the abstract.
8	Randomization	How participants were allocated to interventions.	The method used to allocate participants to the study groups is described (for example, computer-generated sequence, random number table, block randomization). A statement that participants were "randomized", without describing how, was scored 0.
9	Blinding (masking)	Whether or not participants, caregivers and those assessing the outcomes were blinded to group assignment.	The abstract states anywhere in its text that participants or investigators were blinded to group assignment. See note (b) below.
10	Numbers randomized	Number of participants randomized to each group.	The number of participants allocated to each study group is stated. A total sample size alone, without the number in each group, was scored 0.
11	Recruitment	Trial status.	The abstract states the status of the trial — that recruitment is ongoing, that the trial has been completed, or that an interim analysis was performed.
12	Numbers analysed	Number of participants analysed in each group.	The number of participants in each group included in the analysis of the primary outcome is stated.
13	Outcome (results)	For the primary outcome, a result for each group and the estimated effect size and its precision.	The result for the primary outcome is given for each group together with an estimate of the effect size and its precision (for example, risk ratio, odds ratio or mean difference, with a confidence interval). A statement of statistical significance alone, or a p-value alone, was scored 0.
14	Harms	Important adverse events or side effects.	Adverse events or side effects are reported for at least one study group, or the abstract explicitly states that no adverse events or side effects occurred. See note (c) below.
15	Conclusions	General interpretation of the results.	A general interpretation of the results is stated.
16	Trial registration	Registration number and name of trial register.	Both the name of the register and the registration number are stated (for example, ClinicalTrials.gov, NCT…). A register without a number, or a number without a register, was scored 0.
17	Funding	Source of funding.	The source of funding for the trial is stated.

3.1. Notes on the application of three items

(a) Item 2 (Authors). The CONSORT-A item refers to contact details for the corresponding author. In our assessment the item was scored 1 when a contact e-mail address was available for any author of the record, which is a slightly more permissive criterion than the checklist wording.

(b) Item 9 (Blinding). The item was scored 1 only when the abstract reported that participants or investigators had been blinded to group assignment. Abstracts describing the trial as open-label were scored 0, on the reasoning that no blinding had taken place, as were abstracts that made no mention of the matter. We note that the CONSORT-A item is worded as "whether or not" participants, caregivers and outcome assessors were blinded, so that under a literal reading an explicit statement of open-label design would satisfy the item. Our criterion is therefore more demanding than the checklist wording, and the reporting rate we present for this item is correspondingly lower than it would be under a literal application.

(c) Item 14 (Harms). The item was scored 1 both when adverse events or side effects were reported for at least one group and when the abstract explicitly stated that none had occurred, since either statement provides the reader with the information the item is intended to convey. Abstracts that made no mention of harms were scored 0.

4. Study characteristics

The following characteristics were recorded for each included abstract in addition to the checklist items. As with the checklist items, all were coded from the abstract text alone; a code of 0 therefore denotes that the characteristic was not stated.

Characteristic	Coding rule
Number of authors	Recorded as a count, taken from the MEDLINE/PubMed record.
Year of publication	Recorded as the year of publication shown in the MEDLINE/PubMed record.
Journal	Recorded as the journal name shown in the MEDLINE/PubMed record.
Journal impact factor	Recorded from a single, most recent Journal Citation Reports release, not from the release corresponding to the year of publication, because quartile rankings are not available for the earlier part of the study period.
Journal quartile	Recorded from the same Journal Citation Reports release as the impact factor. Journals not indexed were left blank and are reported as “not assigned”.
Type of intervention	1 = pharmacological, where the effect of any medicinal product was tested; 0 = non-pharmacological, for example a surgical procedure, exercise, footwear, dressing, education or a monitoring device.
Intervention category	Free-text description of the intervention, subsequently grouped into ten mutually exclusive categories (Table 7 of the manuscript).
Multicentre design	1 = the abstract states that the trial was conducted at more than one centre; 0 = not stated.
Number of participants	1 = the total number of participants stated in the abstract is 100 or more; 0 = 99 or fewer.
Claimed benefit	1 = the abstract states in the results or the conclusions that the intervention was superior to the comparator; 0 = not claimed.
Declared funding source	1 = the abstract states that the trial was funded by industry; 0 = a non-industry funder (university, non-profit organisation, public body) is stated, or no funding source is stated.
Hospital setting	1 = the abstract states that the trial was conducted in a hospital setting; 0 = not stated.
Abstract structure	1 = the abstract is structured, that is, divided under explicit headings such as Objective, Methods, Results and Conclusions; 0 = unstructured, continuous text.

5. Eligibility and exclusion

Records retrieved by the search were screened on title and abstract. A record was excluded if it did not report a randomized controlled trial with a distinct control group, if diabetic foot was not the primary clinical focus, if no abstract was available, or if the study was not conducted in humans. Excluded records were marked with the reason for exclusion and retained in the working dataset.

6. Assessment procedure and consensus

Each abstract was assessed independently by two reviewers, an endocrinology specialist with clinical trial experience and a research methodologist whose previous work focused on CONSORT compliance. Both applied the written rules set out above throughout the assessment. No separate calibration exercise was carried out before formal scoring; instead, agreement was quantified after independent scoring and any disagreement was resolved by consensus. Inter-rater agreement was calculated on the two independent pre-consensus datasets using Cohen’s κ for each item separately and interpreted according to the categories of Landis and Koch. Discrepancies remaining after independent scoring were resolved through structured discussion, arbitrated where necessary by a third senior investigator experienced in both RCT execution and reporting standards. The dataset used in the published analysis is the post-consensus dataset.

7. Reference

Hopewell S, Clarke M, Moher D, Wager E, Middleton P, Altman DG, Schulz KF; CONSORT Group. CONSORT for reporting randomised trials in journal and conference abstracts. Lancet. 2008;371(9609):281–283.

Hopewell S, Clarke M, Moher D, Wager E, Middleton P, Altman DG, Schulz KF; CONSORT Group. CONSORT for reporting randomized controlled trials in journal and conference abstracts: explanation and elaboration. PLoS Med. 2008;5(1):e20.

Landis JR, Koch GG. The measurement of observer agreement for categorical data. Biometrics. 1977;33(1):159–174.

Supplementary Material S2

STROBE checklist for cross-sectional studies, adapted for methodological research

Evaluating the Quality of Reporting in Randomized Controlled Trial Abstracts on Diabetic Foot: A Cross-Sectional Analysis

The STROBE statement was developed for observational studies of human participants. The present study is a methodological (meta-research) cross-sectional study in which the units of observation are published abstracts rather than individuals, so several items have no direct counterpart. Following the recommendation to adapt reporting guidelines to the study at hand, such items are marked “not applicable” with a brief explanation. Line numbers refer to the revised manuscript.

Section / topic	Item	STROBE recommendation	Line(s)	Where addressed / comment
Title and abstract	1	(a) Indicate the study’s design with a commonly used term in the title or the abstract	2, 20	The title identifies the study as “A Cross-Sectional Analysis”; the abstract states “A cross-sectional observational study was designed”.
		(b) Provide in the abstract an informative and balanced summary of what was done and what was found	17–35	The abstract states the objective, design, sample, scoring instrument, statistical approach, the primary result (median 6 of 17 items, 35.29%), the item-level findings, the temporal comparison and the segmented regression result.
**Introduction**				
Background / rationale	2	Explain the scientific background and rationale for the investigation being reported	41–83	Burden of diabetic foot disease, the growing and heterogeneous trial literature, and the need for clinicians to appraise study quality from the abstract.
Objectives	3	State specific objectives, including any prespecified hypotheses	91-97	The aim is stated at the end of the Introduction. A prespecified hypothesis is not stated separately.
**Methods**				
Study design	4	Present key elements of study design early in the paper	100-106	Cross-sectional observational design, data source and eligibility are presented in the first paragraph of the Methods.
Setting	5	Describe the setting, locations, and relevant dates, including periods of recruitment, exposure, follow-up, and data collection	111-121	MEDLINE/PubMed as the single data source; the search string; the covered period 1994–2026 and the reason the sample cannot extend earlier. Recruitment, exposure and follow-up do not apply, as the units of observation are published abstracts.
Participants	6	(a) Give the eligibility criteria, and the sources and methods of selection of participants	100-127	Eligibility and exclusion criteria for abstracts, the search syntax, and the numbers screened and excluded with reasons.
Variables	7	Clearly define all outcomes, exposures, predictors, potential confounders, and effect modifiers	130-156, 159-190	Outcome: total CONSORT-A score (0–17), also expressed as a percentage of the maximum. Predictors: the study characteristics listed in Section 2.2 and Table 2. Publication year was included as a potential confounder. Item-level definitions are given in Supplementary Material S1.
Data sources / measurement	8	For each variable of interest, give sources of data and details of methods of assessment	130-156	All variables were coded from the abstract text displayed in MEDLINE/PubMed by two independent assessors using a written scoring manual (Supplementary Material S1). Impact factor and journal quartile were taken from a single Journal Citation Reports release, as stated.

Bias	9	Describe any efforts to address potential sources of bias	130-156, 159-190, 390-404	Duplicate independent assessment with κ reported per item and consensus arbitration; scoring restricted to the abstract to avoid contamination from the full text; publication year included as a covariate to address confounding of impact factor and quartile with time; segmented regression used to separate a guideline effect from the secular trend. Residual sources of bias are discussed in Section 4.1.
Study size	10	Explain how the study size was arrived at	123-127	The sample comprises all records retrieved by the search that met the eligibility criteria (606 of 746). No sampling was performed and no a priori sample size calculation was applicable.
Quantitative variables	11	Explain how quantitative variables were handled in the analyses. If applicable, describe which groupings were chosen and why	159-190, Table 2	The total score was analysed on the percentage-of-maximum scale. Number of authors and impact factor were dichotomised at their median values; number of participants was dichotomised at 100. Category boundaries are stated in Tables 2, 5 and 6.
Statistical methods	12	(a) Describe all statistical methods, including those used to control for confounding	159–190	Descriptive statistics; Cohen's κ with Landis and Koch interpretation; Mann-Whitney U test; univariate and pre-specified multivariable linear regression with HC3 robust standard errors; publication year as a covariate; variance inflation factors.
		(b) Describe any methods used to examine subgroups and interactions	159–190	No subgroup or interaction analyses were performed. Item-level comparisons between the two publication periods were made by the χ^2 test with Benjamini-Hochberg correction across the 17 items.
		(c) Explain how missing data were addressed	159–190	Journal quartile could not be assigned for two journals; analyses involving quartile are based on 604 abstracts and all others on 606. Characteristics not stated in an abstract were coded as not stated rather than treated as missing.
		(d) If applicable, describe analytical methods taking account of sampling strategy	—	Not applicable: the full eligible population of retrieved records was analysed, so no sampling weights or design effects arise.
		(e) Describe any sensitivity analyses	159–190, 266–275	Segmented regression around 2008 and repetition of the dichotomous comparison at alternative cut-points. A Poisson model for the 0–17 count was fitted as a further check and gave the same pattern of associations.
**Results**				
Participants	13	(a) Report numbers of individuals at each stage of study	123-127	746 records retrieved; 140 excluded; 606 abstracts analysed.
		(b) Give reasons for non-participation at each stage	123-127	Reasons for exclusion are reported: 98 not a randomized controlled design, 22 diabetic foot not the primary topic, 20 no abstract available.
		(c) Consider use of a flow diagram	—	A flow diagram is not included. A PRISMA-type diagram is reserved for systematic reviews, and the numbers at each stage are reported in full in the text.
Descriptive data	14	(a) Give characteristics of study participants and information on exposures and potential confounders	199-206	Characteristics of the included abstracts are given in Table 2, with mean impact factor and number of authors.
		(b) Indicate number of participants with missing data for each variable of interest	207, Table 2	Table 2 reports the two abstracts for which journal quartile could not be assigned.
Outcome data	15	Report numbers of outcome events or summary measures	209-226, Tables 3 and 4	Reporting rate for each of the 17 items (Table 3) and summary measures of the total score (Table 4).
Main results	16	(a) Give unadjusted estimates and, if applicable, confounder-adjusted estimates and their precision. Make clear which confounders were adjusted for and why	230-252, Table 6	Univariate and multivariable estimates with 95% confidence intervals are given in Table 6. The multivariable model contains all study

				characteristics and publication year; the legend states that estimates are in percentage points and gives the adjusted R^2 and n.
		(b) Report category boundaries when continuous variables were categorized	159-190, Tables 2, 5 and 6	Median cut-points for number of authors (6) and impact factor (2.60) and the 100-participant boundary are stated.
		(c) If relevant, consider translating estimates of relative risk into absolute risk	—	Not applicable: the outcome is a continuous score and estimates are already reported on an absolute scale (percentage points).
Other analyses	17	Report other analyses done	266-295	Segmented regression around 2008; the dichotomous comparison at alternative cut-points; item-level comparison between periods (Table 8); and the proportion of abstracts claiming benefit without a supporting effect estimate.
**Discussion**				
Key results	18	Summarise key results with reference to study objectives	305-320, 325-341	Overall adherence, the worst-reported items and the temporal findings are summarised at the start of the Discussion.
Limitations	19	Discuss limitations of the study, taking into account sources of potential bias or imprecision	370-389	Section 4.1 sets out five limitations: the 1994 MeSH constraint on the pre-2008 period, evaluation of abstracts alone, coding of characteristics from abstracts, attribution of impact factor and quartile, and restriction to PubMed.
Interpretation	20	Give a cautious overall interpretation of results considering objectives, limitations, multiplicity of analyses, results from similar studies, and other relevant evidence	325-377	The Discussion compares the findings with Ghimire et al., Germini et al., Hopewell et al., Song et al., Vrebalov Cindro et al. and Vucinovic et al., and interprets the regression findings as properties of reporting. Multiplicity across the 17 item-level comparisons was addressed by the Benjamini-Hochberg procedure.
Generalisability	21	Discuss the generalisability (external validity) of the study results	370-389	Section 4.1 notes that the sample was restricted to RCTs indexed in PubMed, so studies in other databases or the grey literature may not have been captured.
**Other information**				
Funding	22	Give the source of funding and the role of the funders for the present study	426	The Funding statement declares that the research received no external funding.

Reference: von Elm E, Altman DG, Egger M, Pocock SJ, Gøtzsche PC, Vandenbroucke JP; STROBE Initiative. The Strengthening the Reporting of Observational Studies in Epidemiology (STROBE) statement: guidelines for reporting observational studies. Lancet. 2007;370(9596):1453–1457.
